# Clinical outcomes following a traditional Chinese medicine foot bath preparation for lower-limb edema and sleep disturbance: a single-center retrospective study of 2,000 patients

**DOI:** 10.3389/fphar.2026.1884794

**Published:** 2026-09-22

**Authors:** Yehua Zhao, Jingwang Wei, Shanbairong Nie, Chanyuan Zhang, Dan Wang

**Affiliations:** 1 Department of Gastroenterology, The Second People’s Hospital of Lanzhou City, Lanzhou, China; 2 Department of Neurosurgery, The Second People’s Hospital of Lanzhou City, Lanzhou, China; 3 Hamilton College, Clinton, NY, United States; 4 Department of Geriatrics, The Second People’s Hospital of Lanzhou City, Lanzhou, China

**Keywords:** blood activation and stasis resolution, lower limb edema, retrospective observational study, sleep disorders, traditional Chinese medicine foot bath

## Abstract

**Objective:**

To evaluate clinical outcomes and documented adverse events following treatment with a traditional Chinese medicine (TCM) foot bath preparation in patients with lower-limb edema and sleep disturbance.

**Methods:**

This single-center retrospective observational study included 2,000 patients treated at Lanzhou Second People’s Hospital between May 2017 and May 2022. All patients had varying degrees of lower-limb edema, peripheral circulatory dysfunction, and sleep disturbance. Patients received a standardized TCM foot bath preparation consisting of stir-fried peach kernel (10 g), bush cherry seed (15 g), Chinese angelica root (10 g), and safflower (10 g). The preparation was administered once daily for 20 sessions, comprising two consecutive 10-day treatment courses. Clinical outcomes were assessed by comparing ankle circumference, ABI, and PSQI values before and after treatment.

**Results:**

Using the routine record-based classification, 1,120 patients (56.0%) were classified as “markedly effective,” 770 (38.5%) as “effective,” and 110 (5.5%) as “ineffective.” The combined proportion was 94.5%; however, this classification is not internationally validated and is reported descriptively rather than as evidence of effectiveness. After treatment, ankle circumference and PSQI scores were significantly lower than baseline values (both P < 0.01), whereas ABI values were significantly higher (P < 0.05). Mild local adverse reactions, including transient redness and dryness, were observed in a small proportion of patients.

**Conclusion:**

In this single-center retrospective observational study, post-treatment changes were observed in lower-limb edema, peripheral circulation, and sleep quality.

## Introduction

Oedema of the lower limbs and sleep disorders are frequently encountered clinical issues which significantly impact physical function, psychological wellbeing, and quality of life, particularly in patients of the middle and elderly age groups (Olinic et al., 2018; Patel et al., 2018). Oedema in the lower limbs is due to an abnormal increase in interstitial fluid, which can be caused by a reduction in venous return, blockage of lymphatics, increased capillary permeability and peripheral microcirculatory abnormalities (Dormandy and Rutherford, 2000). The symptoms can include swelling, heaviness, soreness, numbness, fatigue, restricted movement, and ongoing discomfort in the lower limbs (Olinic et al., 2018). Autonomic nervous system imbalance, inflammation, endocrine disturbance and psychological stress are all linked to sleep disorders such as insomnia, delayed sleep onset, fragmentation and poor sleep quality (Irwin and Opp, 2017; Irwin, 2015). There is also evidence that poor sleep quality may exacerbate endothelial dysfunction, sympathetic activation, inflammatory cytokine release and peripheral circulatory impairment (Ja et al., 2017). On the other hand, pain, vascular congestion, oedema and discomfort in lower limbs may affect the initiation and maintenance of sleep, which in turn may affect the circulation (Mullington et al., 2009). Patients with both conditions tend to have fatigue, anxiety, irritability, depression, and decreased quality of life, and therapeutic targets should attempt to enhance peripheral circulation and to regulate sleep (Krystal and Edinger, 2008).

Treatment for lower limb oedema currently includes diuretics, compression therapy, elevating limbs, exercise, physiotherapy and treating any underlying systemic diseases (Dormandy and Rutherford, 2000). These techniques can help alleviate symptoms, but in the long term, they can be ineffective because of recurrence, their inability to be used consistently or side effects, including electrolyte imbalance, dehydration, skin irritation, dizziness and discomfort. Likewise, drug treatment for insomnia can lead to tolerance and dependence, as well as an effect on cognition, daytime sleepiness and remaining fatigue (Patel et al., 2018). Hence, for patients with chronic oedema and sleep disturbances, there is a need for safe, economical, minimally invasive and holistic interventions.

Traditional Chinese Medicine (TCM) offers an integrative approach to therapy for oedema, vascular dysfunction and insomnia based on syndrome differentiation and external therapies (Yang et al., 2022). According to the theory of TCM, oedema is primarily associated with the dysfunction of the spleen, kidney and lung systems, which results in dysfunction of the body fluids, retention of dampness, stagnation of Qi and blood stasis in the meridians (Cheng et al., 2017). Yin-Yang disharmony, insufficient blood nourishment of the heart, disturbance of the Shen, internal heat or Qi and blood stagnation are common etiologies associated with sleep disorders (Su et al., 2021). As the smooth flow of Qi and blood is a precondition for the balance of both body and mind, therapies that activate blood flow, resolve stasis, eliminate dampness and calm the mind can be simultaneously used to reduce oedema and improve sleep quality.

TCM foot bath preparation is a widely used external therapy in TCM due to its simple application, safety, convenience, and clinical applicability (Tia et al., 2024). This therapy is a combination of the warm water thermal effect, the botanical transdermal effect and the foot acupoint and meridian stimulation effect. TCM theory suggests that the feet are closely related to the three Yin and three Yang meridians, and there are an abundance of capillaries, nerve endings, and reflex zones in the feet (Su et al., 2021). Warm botanical immersion can help to stimulate peripheral vasodilation, enhance blood flow, increase venous return, relax muscles and boost local metabolism (Charkoudian, 2010). Foot acupoint stimulation can also help to regulate the autonomic nervous system, decrease sympathetic overactivity, alleviate stress and induce sleep (Wen et al., 2020). More recent research also indicates that warm-water immersion and active ingredients in botanical drugs can impact microcirculation, endothelial function, vascular resistance, inflammation, oxidative stress, pain and sedation pathways (Yang et al., 2022). Clinical evidence has shown that TCM foot bath preparation is beneficial for diabetes peripheral neuropathy, chronic venous insufficiency, fatigue syndromes, insomnia, rehabilitation and chronic pain disorders (Song et al., 2023; Yang et al., 2010). There is, however, a lack of large-scale retrospective studies which have assessed its combined effect on lower limb oedema and sleep disorders.

The botanical formula used in this research includes the following botanical drugs: stir-fried peach kernel, bush cherry seed, Chinese angelica and safflower. This prescription is based on the principle of activating blood circulation, resolving blood stasis, promoting diuresis, nourishing blood and calming the Shen in TCM. Peach kernel and safflower have traditionally been used for the promotion of circulation and elimination of blood stasis, and Chinese angelica nourishes and invigorates blood. Bush cherry seed is frequently used for diuretics, to remove dampness and for anti-oedema (Su et al., 2021). This therapy, due to its botanical, thermal and meridian activation effects, can help alleviate venous congestion, increase peripheral circulation, decrease interstitial fluid, relieve discomfort in the lower limbs, and aid in sleep improvement (Chang et al., 2025).

This retrospective study aimed to assess the clinical efficacy and safety of one traditional Chinese medicine (TCM) foot bath preparation for patients with lower limb oedema and sleep disorders. A total of 2,000 patients were treated between May 2017 and May 2022 at Lanzhou Second People’s Hospital, and their clinical data were analyzed. Ankle circumference, Ankle-Brachial Index (ABI) and Pittsburgh Sleep Quality Index (PSQI) scores were used to evaluate treatment outcomes before and after therapy (Abraham et al., 2012; Buysse et al., 1989). This large sample retrospective observational study might give some real clinical evidence on the application of external therapy of TCM in rehabilitation medicine and chronic disease management.

## Materials and methods

### Study design and ethics concerns

This retrospective clinical study was carried out in Lanzhou Second People’s Hospital. Analysis of medical records of patients who were treated with Traditional Chinese Medicine (TCM) foot bath preparation between May 2017 and May 2022 was included. The objective of this study was to evaluate the efficacy of a standardized TCM foot bath preparation in the improvement of lower limb oedema, peripheral blood circulation, and sleep quality of patients with chronic lower limb circulatory dysfunction with sleep disorders. The study protocol was carried out in accordance with the ethical principles of the Declaration of Helsinki and institutional guidelines for clinical research. Patient confidentiality and anonymity were strictly preserved during data collection and analysis. This was a retrospective investigation using analysis of routinely collected clinical data, and the institutional review committee waived the informed consent requirements. No patient identifying information was disclosed during the preparation of the manuscript or the statistical analysis. As this was a retrospective uncontrolled observational study, no randomization, blinding, placebo intervention, or standard-care control group was applied; therefore, the study was designed to describe treatment-associated clinical changes rather than establish causal efficacy.

### General clinical information

The clinical data of 2,000 patients receiving treatment in Lanzhou Second People’s Hospital during the study period were collected retrospectively. The study population consisted of 856 men and 1,144 women aged between 35 and 78 years, with a mean age (SD) of 56.4 ± 10.2 years. Disease duration was between 6 months and 10 years. All patients had different degrees of soreness, swelling, circulatory disorder, non-pitting or mild pitting oedema of the lower limbs, difficulty falling asleep, difficulty maintaining sleep or easy waking during the night. The majority of patients reported chronic symptoms that negatively affected physical activity, daily functioning, emotional stability and quality of life. Most patients reported aggravation of symptoms following prolonged standing, inactivity, fatigue or change of season temperature. In many patients, previous conventional therapies such as limb elevation, oral medications, massage or physiotherapy provided only temporary or limited improvement. Because this retrospective analysis was based on existing clinical records, concurrent medications, compression therapy, physiotherapy, massage, limb elevation, exercise habits, dietary factors, and other lifestyle-related variables could not be fully standardized or adjusted. These factors may have influenced edema severity, peripheral circulation, and sleep quality and were considered potential confounders.

### Diagnostic criteria

The study patients were diagnosed with “Oedema” and “Insomnia” based on the Criteria for Diagnosis and Therapeutic Effect of Diseases and Syndromes in Traditional Chinese Medicine. The diagnostic criteria used in this retrospective study were based on clinical symptoms, physical examination findings, and Traditional Chinese Medicine-related diagnostic standards recorded in routine clinical practice. Sleep disorder diagnosis was supported by documented sleep-related symptoms and assessment using the Pittsburgh Sleep Quality Index (PSQI), which was used as the structured instrument for evaluating sleep quality in this retrospective dataset. We acknowledge that these criteria are not fully equivalent to internationally standardized diagnostic classifications for oedema or sleep disorders, which should be considered when interpreting the findings.

The diagnostic criteria for lower limb oedema were:

- Continued swelling or enlargement of the lower extremities;
- Heaviness, soreness, tightness or tiredness in the legs;
- Presence of non-pitting or mild pitting oedema;
- Gets worse with prolonged standing or activity;
- Poor circulation in the periphery or coldness of the lower extremities.

The diagnostic criteria of a sleep disorder were as follows:

- Difficulty falling asleep;
- Frequent nocturnal awakening;
- Reduced sleep duration;
- Low quality of sleep;
- Daytime fatigue, irritability, and impaired concentration associated with insufficient sleep.

### Inclusion and exclusion criteria

#### Criteria for inclusion

Patients were eligible if they met all of the following criteria:

- Diagnosed with chronic oedema of the lower limbs and sleep disorders;
- Between 35 and 78 years old;
- Completed the full treatment protocol consisting of two consecutive courses, with 10 once-daily sessions per course and 20 sessions in total;
- Had detailed clinical records and follow-up data;
- Pre-treatment and post-treatment assessment of ankle circumference, ABI and PSQI scores.

#### Criteria for exclusion

Exclusions for patients were:

- Severe heart failure or kidney failure;
- Acute deep-vein thrombosis;
- Severe occlusive peripheral arterial disease;
- Skin ulceration, active infection or severe dermatitis of the lower limbs;
- Pregnancy and lactation.
- Serious mental disorders;
- Missing medical records or interrupted treatment courses.

Therefore, the final analysis included only patients with complete pre-treatment and post-treatment assessments for ankle circumference, ABI, and PSQI, and no imputation was performed for missing outcome data.

### Botanical preparation reporting and ConPhyMP compliance

The botanical foot bath preparation used in this retrospective study was reviewed and documented according to the ConPhyMP statement and the Good Practice in the Research and Publication of Medicinal Plant and Botanical Medicine Studies recommendations. The relevant ConPhyMP tables for botanical preparations, including Table 1 and Supplementary Table S1, were completed and provided as a supplementary PDF file with the revised submission. The preparation was reported with attention to botanical drug identity, taxonomic validation, plant part used, authentication, voucher documentation, preparation procedure, drug–extract ratio, pharmaceutical parameters, and quality-control requirements.

**TABLE 1:** Composition, botanical identity, taxonomy, authentication, and source documentation of the traditional Chinese medicine foot bath preparation.

Botanical drug (Chinese name)	Pharmacopoeial drug name	Botanical source(s)	Family	Plant part/drug material	Processing status	Quantity	Reported quality marker(s)	Source and authentication documentation
Stir-fried peach kernel(Tao Ren)	Persicae Semen	*Prunus persica (L.) Batsch; Prunus davidiana (Carrière) Franch*	Rosaceae	Dried mature seed/kernel	Stir-fried/processed; dry-fried to the specified pharmacopeial appearance and odor, then cooled	10 g	Amygdalin; related cyanogenic glycosides, fatty acids, flavonoids, phenolic acids, and volatile constituents	Supplier: Lanzhou Antaitang Traditional Chinese Medicine Pieces Co., Ltd. Batch: 25090513 Authentication no.: Gan 20160145 Authenticated by: Wang Yixing Voucher: T001200106 Depository: Traditional Chinese Medicine Warehouse, Second People’s Hospital of Lanzhou City
Bush cherry seed(Yu Li Ren)	Pruni Semen/Semen Pruni	*Prunus humilis Bunge; Prunus japonica Thunb.; Prunus pedunculata (Pall.) Maxim*	Rosaceae	Dried mature seed	Crude dried seed used for aqueous decoction unless additional processing is documented	15 g	Amygdalin; prunasin and other cyanogenic glycosides; fatty oils, sterols, flavonoids, and phenolic metabolites	Supplier: Lanzhou Antaitang Traditional Chinese Medicine Pieces Co., Ltd. Batch: 23060301 Authentication no.: Gan 20160145 Authenticated by: Wang Yixing Voucher: T000300863 Depository: Traditional Chinese Medicine Warehouse, Second People’s Hospital of Lanzhou City
Chinese angelica root(Dang Gui)	Angelicae Sinensis Radix	*Angelica sinensis (Oliv.) Diels*	Apiaceae	Dried root	Crude dried root used for aqueous decoction unless sliced or otherwise processed material is documented	10 g	Ferulic acid; Z-ligustilide; other phthalides, phenolic acids, polysaccharides, organic acids, and volatile constituents	Supplier: Gansu Longmai Medicinal Materials Co., Ltd. Batch: 2605044 Authentication no.: Gan 20160020 Authenticated by: Guo Zhongfang Voucher: T001700173 Depository: Traditional Chinese Medicine Warehouse, Second People’s Hospital of Lanzhou City
Safflower(Hong Hua)	Carthami Flos	*Carthamus tinctorius L*	Asteraceae	Dried flower/floret	Crude dried floret used for aqueous decoction	10 g	Hydroxysafflor yellow A; safflor yellow pigments, carthamin, quinochalcone glycosides, flavonoids, and phenolic metabolites	Supplier: Hebei Mingzhu Pharmaceutical Co., Ltd. Batch: 25120801 Authentication no.: Ji 20160039 Authenticated by: Guo Zhongfang Voucher: T001200349 Depository: Traditional Chinese Medicine Warehouse, Second People’s Hospital of Lanzhou City

### Protocol of treatment

#### Botanical drug composition and preparation

The botanical foot bath preparation consisted of four botanical drug materials used in fixed quantities for all patients. The composition, botanical identity, taxonomy, plant part, processing status, quantity, authentication, and source documentation of each botanical drug are summarized in Table 1. The same formulation and preparation procedure were applied throughout the treatment period to maintain consistency in clinical use. The botanical drug materials were documented using botanical nomenclature, author citations, family names, medicinal plant parts, and pharmacopoeial drug names where applicable.

The TCM foot bath preparation used in this study was:

The formula was developed following the TCM therapeutic principles of activating blood circulation and resolving blood stasis, promoting urination to reduce oedema, and nourishing blood to calm the Shen (mind).

### Preparation of decoction

The botanical drugs were placed in a cloth bag and soaked in 2,000 mL of water for 30 min before decoction. The mixture was initially boiled on high heat and then simmered for approximately 20 min until there was almost 1,500 mL of medicinal decoction left. Then the prepared decoction was poured into a basin for a foot bath and diluted with warm water to a final therapeutic temperature of about 40 °C–42 °C.

### Quality control, pharmaceutical parameters, and chemical profiling of the botanical preparation

The botanical drug materials were used according to a fixed prescription and standardized preparation procedure. For each preparation, the total amount of crude botanical drug material was 45 g, consisting of stir-fried peach kernel, bush cherry seed, Chinese angelica, and safflower in fixed quantities. The materials were soaked in 2000 mL of water for 30 min, decocted, and concentrated to approximately 1,500 mL before dilution for external foot bath use. Therefore, the crude botanical drug-to-decoction ratio was 45 g:1,500 mL, corresponding to an approximate crude botanical drug concentration of 30 mg/mL. The decoction was prepared fresh for external use and administered at approximately 40 °C–42 °C.

The quality-control framework was documented according to pharmacopoeial and ConPhyMP-based reporting requirements. The botanical identity, medicinal part, botanical drug name, preparation procedure, final decoction volume, drug-to-decoction ratio, and basic pharmaceutical parameters are summarized in the Supplementary Table S1. For chemical-profile reporting, complementary quality-control approaches were specified, including macroscopic/micro scopic identification, chromatographic fingerprinting, and marker-metabolite profiling. The reference metabolites considered for quality-control documentation included amygdalin for Persicae Semen and Pruni Semen, ferulic acid and ligustilide-related metabolites for Angelicae Sinensis Radix, and hydroxysafflor yellow A for Carthami Flos.

### Foot bath treatment

The patients were instructed to sit in a relaxed and comfortable position with both feet immersed in the botanical solution up to the level of the Sanyinjiao (SP6) acupoint, which is located on the lower one-third of the calf. Each treatment session was approximately 20–30 min. Additional warm water was added as needed to maintain a stable therapeutic temperature during treatment. The treatment was administered once daily. One treatment course consisted of 10 daily foot bath sessions, and all patients completed two consecutive courses without a planned interval between courses. Therefore, the full treatment protocol included 20 daily sessions in total, lasting approximately 20 days. Patients were advised not to be exposed to a cold environment immediately after the treatment, and sufficient rest and water intake were advised during treatment.

The overall treatment workflow and outcome assessment procedure are illustrated in Figure 1.

**FIGURE 1:**
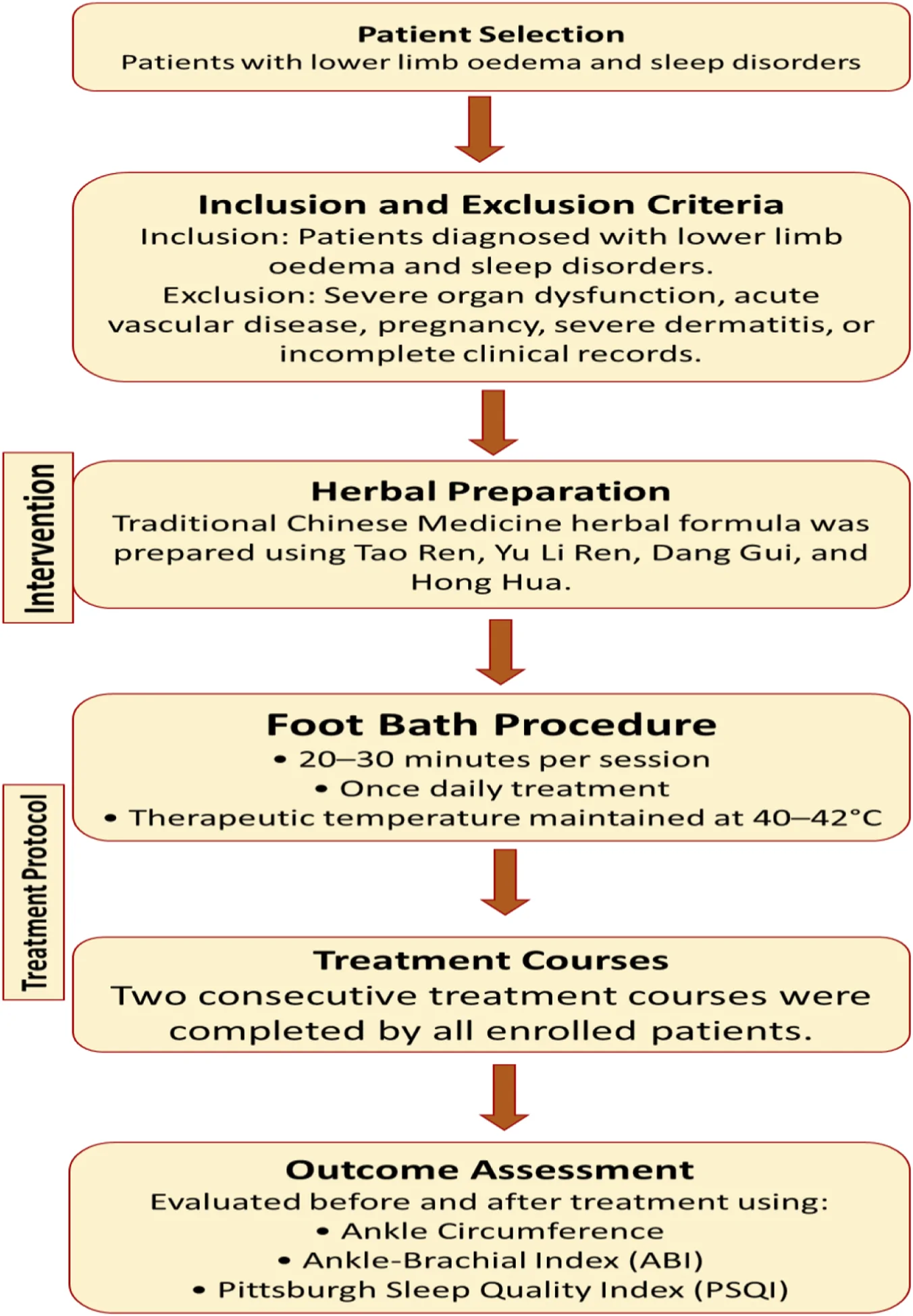
Treatment workflow of Traditional Chinese Medicine (TCM) foot bath preparation. The figure illustrates the stepwise clinical workflow used in the present retrospective study, including patient screening, eligibility assessment, botanical preparation, daily foot bath intervention, treatment course completion, and evaluation of clinical outcome measures, including ankle circumference, Ankle-Brachial Index (ABI), and Pittsburgh Sleep Quality Index (PSQI).

### Measures of outcome

The main quantitative outcome measures were ankle circumference, Ankle-Brachial Index (ABI), and Pittsburgh Sleep Quality Index (PSQI) (Table 2). ABI and PSQI were chosen due to their good clinical implementation as markers of peripheral circulation and sleep quality, respectively. Ankle circumference was adopted as an acceptable surrogate clinical measurement of lower limb swelling for daily use in routine practice. Still, it is not a recognized universal diagnostic point for the severity of swelling. Baseline assessment was carried out before the first foot bath treatment on the day of treatment under normal clinical conditions with the patient resting for around 15 min. Assessment of post-treatment occurred within 24 h of the end of the second treatment. Outcome measures were not obtained as time series following each daily session, but rather at two consistent time points: baseline and end of the entire treatment protocol (ankle circumference, ABI, PSQI). All patients included in this assessment schedule were assessed in the same manner and patients with incomplete or non-standard assessment timing were excluded from the final analyses. To address the need for objective assessment, the traditional symptom-based indications were translated into measurable clinical outcomes before analysis. Lower-limb swelling was evaluated using standardized ankle circumference measurement, peripheral circulation was evaluated using ABI, and sleep disturbance was evaluated using PSQI. Thus, although the intervention was selected according to Traditional Chinese Medicine principles, treatment-associated changes were assessed using predefined quantitative and structured clinical measures rather than relying only on subjective symptom descriptions.

**TABLE 2:** Comparison of outcome measures before and after treatment (x̄ ± s).

Indicator	Before treatment	After treatment	Mean difference	95% CI	Effect size	P-value
Ankle circumference (cm)	24.58 ± 2.14	22.31 ± 1.87	−2.27	−2.39 to −2.15	−1.13	<0.01
ABI	0.89 ± 0.12	0.98 ± 0.10	0.09	+0.083 to +0.097	+0.81	<0.05
PSQI score	14.25 ± 3.12	7.86 ± 2.45	−6.39	−6.56 to −6.22	−2.28	<0.01

The following measures were used to describe pre-treatment and post-treatment clinical outcomes.

### Assessment of lower limb oedema

The severity of swelling in the lower legs was determined by measuring the circumference of the ankles at a standardized level 5 cm above the medial malleolus. Before and after treatment, the measurements were taken in the same anatomical location and in the same conditions with a flexible measuring tape. Ankle circumference was chosen as a readily available bedside measure of local swelling as interstitial fluid accumulation could cause an increase in limb circumference. Thus, a decrease in ankle circumference was considered to be a positive indicator of a decrease in swelling in the lower limbs. Ankle circumference, however, was not considered as an independent internationally validated diagnostic endpoint and was interpreted together with ABI, PSQI and changes in clinical symptoms.

### Evaluation of peripheral circulation

The circulation of peripheral blood was assessed by Ankle-Brachial Index (ABI). ABI was determined by using systolic blood pressure in the ankle and brachial artery according to standard procedures of vascular evaluation. The increase in ABI values after treatment was due to the improvement of peripheral circulation and lower limb arterial perfusion.

### Sleep quality evaluation

Sleep quality was assessed using the Pittsburgh Sleep Quality Index (PSQI), a validated clinical tool commonly used for the assessment of sleep disorders. PSQI scores range from 0 to 21, with higher scores indicating poorer sleep quality. Improvement in sleep quality and sleep-related symptoms was interpreted as a reduction in PSQI scores after treatment.

### Supportive clinical response classification

As part of routine TCM clinical record documentation, treatment response was descriptively classified into three categories according to symptom improvement and changes in clinical measures. This classification was used only as supportive descriptive information and was not treated as a primary validated outcome endpoint.

### Highly effective

Complete or almost complete resolution of oedema of lower limbs. Normalization of the circumference of the ankle. Significant improvement in circulation. Reduction of PSQI score by ≥50%.

### Effective

Partial improvement in oedema and lower limb discomfort, reduction in ankle circumference >1 cm and reduction in PSQI score of 25%–49%.

### Ineffective

No obvious improvement or deterioration of oedema, ankle circumference reduction rate less than 1 cm, and PSQI reduction rate less than 25%.

The total effective rate was determined by:

Total Effective Rate=[(Markedly Effective Cases+Effective Cases)/Total Cases]×100%

The total effective rate is commonly used in some TCM clinical reports as a composite descriptive indicator; however, it is not an internationally validated evidence-based endpoint. Therefore, the total effective rate was de-emphasized in interpretation, and the main outcome evaluation relied on continuous quantitative measures, including ankle circumference, ABI, and PSQI.

### Assessment of safety

Safety was determined through general observations and patient self-reporting at each foot bath treatment. Prior to each session, the skin condition of the lower limbs was assessed for redness, ulceration, infection, dermatitis or any other abnormalities. Patients were observed during therapy for local discomfort, excessive sensation of heat, burning, itching, redness of the skin, dryness, dizziness, allergic reactions and/or thermal injury. Water temperature was kept at around 40 °C–42 °C and special care was taken for the elderly, diabetic and patients with impaired peripheral sensation. The water temperature was adjusted, treatment duration shortened, or the treatment temporarily stopped according to clinical judgment if discomfort or abnormal reactions of the skin occurred. Adverse events were classified according to severity: mild, moderate or severe. Mild adverse events were those that involved only transient local adverse reactions (temporary skin redness, mild dryness, itching, warmth sensation or discomfort) that occurred spontaneously or required only simple supportive care. Moderate adverse events were considered to be those that necessitated interruption of the treatment, topical treatment, medical evaluation or extended observation. Severe adverse events included burns, severe allergic reactions, infection, cardiovascular symptoms, hospitalisation or events that required discontinuation of treatment or significant clinical harm. Safety assessments were conducted during the treatment period. Special attention was paid to elderly and diabetic patients, as they are more susceptible to temperature-related injury.

Potential safety considerations related to the botanical preparation were also considered. Because the formula contains blood-activating botanical drugs, including Persicae Semen and Carthami Flos, caution was applied in patients with bleeding tendency, severe vascular disease, skin lesions, pregnancy, lactation, or use of medications that may increase bleeding risk. Patients with active lower-limb ulceration, infection, severe dermatitis, pregnancy, lactation, severe occlusive peripheral arterial disease, acute deep-vein thrombosis, severe heart failure, or kidney failure were excluded. As the preparation was administered externally as a warm foot bath, local skin irritation, redness, dryness, itching, excessive heat sensation, allergic reaction, and thermal injury were specifically monitored during treatment. Elderly patients, diabetic patients, and patients with impaired peripheral sensation received additional temperature supervision to reduce the risk of heat-related injury.

### Statistical analysis

Standard statistical software was used in statistical analysis. Continuous variables were presented as mean ± standard deviation (x̄ ± s). A paired t-test was used to compare pre- and post-treatment values. Wherever possible, mean differences with 95% confidence intervals were also reported in addition to P-values better illustrate the magnitude and precision of treatment-associated changes. Categorical variables were described by means of frequency and percentage. For the final analysis, only patients with complete baseline and post-treatment information on all three parameters–ankle circumference, ABI, and PSQI–were included. The following exclusion criteria were applied: patients lacking medical records or who had a period of interruption in the treatment course. Since this was a retrospective single-arm study, information regarding concurrent treatment, lifestyle factors and disease severity was not always available, and the full adjustment for potential confounding could not be achieved. The P-values of <0.05 were set as the significant level, and P-values of <0.01 were set as the highly significant level.

## Results

### General clinical course

The final retrospective analysis comprised 2000 patients in total who completed the prescribed TCM foot bath treatment protocol. All patients underwent two consecutive treatment courses without interruption, and complete clinical data before and after treatment were available for statistical assessment. Overall, patients had differing degrees of improvement in lower extremity oedema, peripheral circulation, subjective lower extremity discomfort and sleep quality after treatment.

Among the 2,000 patients included in the analysis, 1,120 (56.0%) were recorded as “markedly effective,” 770 (38.5%) as “effective,” and 110 (5.5%) as “ineffective.” These non-validated categories are reported as descriptive information only. The combined 94.5% proportion should not be interpreted as evidence of treatment effectiveness. The distribution of routine clinical response categories is illustrated in Figure 2.

**FIGURE 2:**
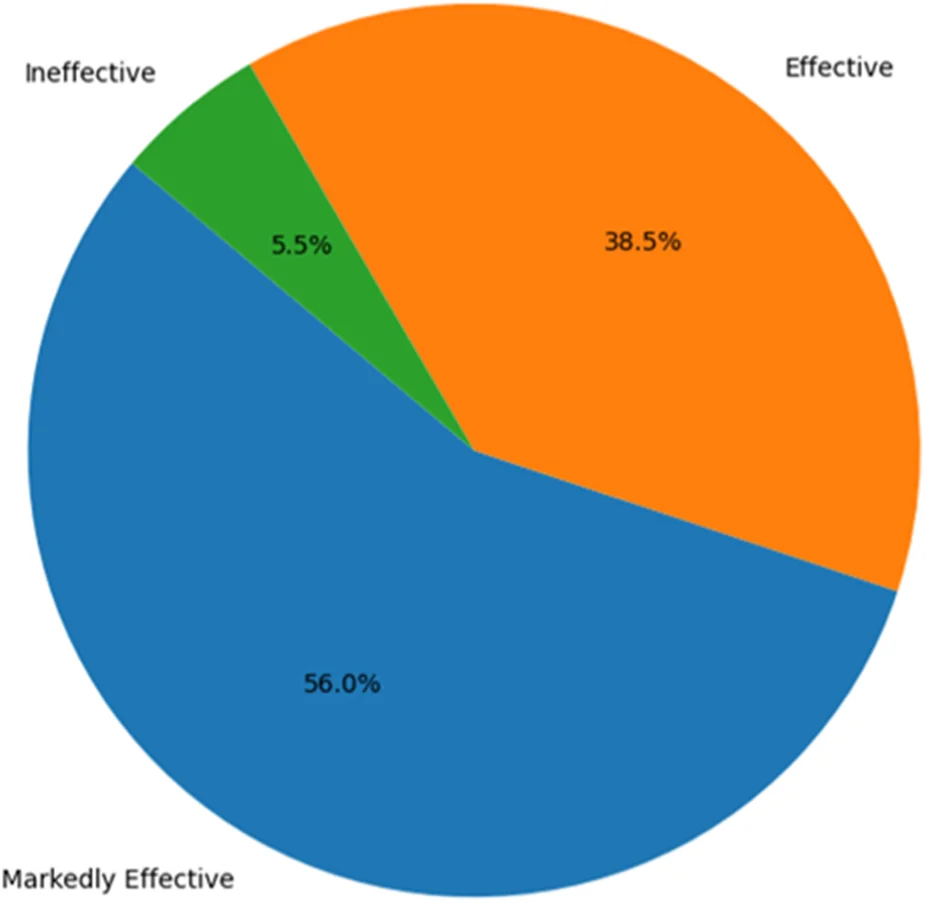
Distribution of routine clinical response categories after treatment with the traditional Chinese medicine foot bath preparation. Among the 2,000 patients included in the analysis, 1,120 (56.0%) were classified as “markedly effective,” 770 (38.5%) as “effective,” and 110 (5.5%) as “ineffective.” The combined proportion of the first two categories was 94.5%. These categories were based on routine clinical records, were not internationally validated, and are presented descriptively; they should not be interpreted as evidence of treatment efficacy or effectiveness.

### Improvement of lower extremity oedema

The lower limb oedema assessment revealed a significant improvement after treatment. Most patients had some degree of ankle swelling, heaviness, soreness, tightness and fatigue of the lower extremities prior to therapy. Some patients also reported that the discomfort was worsened by standing for long periods or by physical activity. Measurements of ankle circumference 5 cm above the medial malleolus post-treatment showed a statistically significant reduction compared to baseline pre-treatment values. The mean ankle circumference was significantly decreased from 24.58 ± 2.14 cm before treatment to 22.31 ± 1.87 cm after treatment (P < 0.01). The reduction in ankle circumference before and after treatment is illustrated in Figure 3.

**FIGURE 3:**
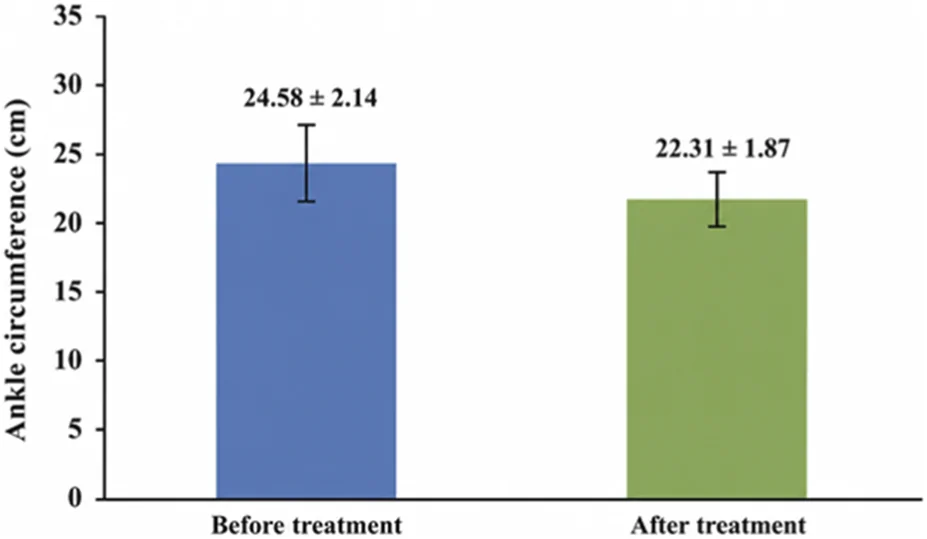
Comparison of ankle circumference before and after TCM foot bath preparation. Error bars represent standard deviation.

This reduction in ankle circumference indicated effective reduction of fluid retention and oedema of the lower extremities. Clinically, patients reported a reduction in feelings of heaviness, greater comfort during walking, improved mobility, and less fatigue in the lower limbs. At the post-treatment assessment conducted after completion of the full 20-session protocol, ankle circumference was lower than the baseline value.

### Improved peripheral blood flow

Peripheral blood circulation was examined using the Ankle-Brachial Index (ABI), a validated measure of lower extremity arterial perfusion and vascular function. Many patients had reduced ABI values, indicating impaired peripheral circulation and microvascular dysfunction before treatment. ABI values were significantly increased after treatment (0.89 ± 0.12 before treatment vs. 0.98 ± 0.10 after treatment, P < 0.05), suggesting significant improvement of lower limb blood flow and vascular circulation. As shown in Figure 4, ABI values significantly improved after treatment.

**FIGURE 4:**
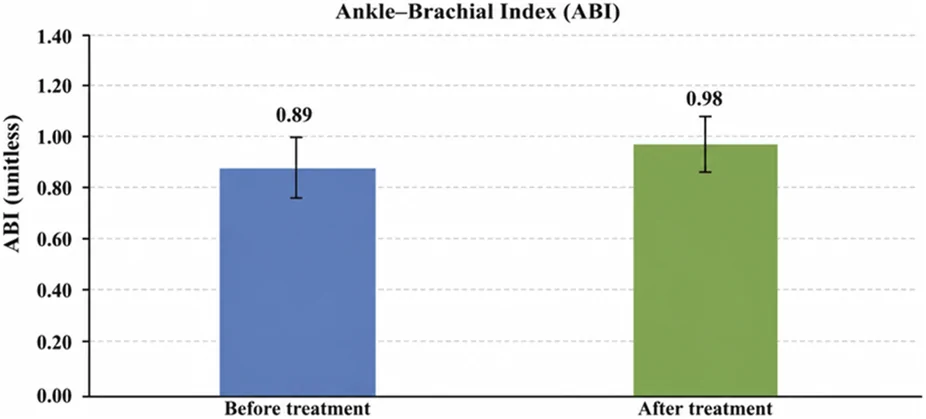
Comparison of Ankle–Brachial Index (ABI) before and after TCM foot bath preparation. Error bars represent standard deviation.

ABI values improved, indicating better perfusion of peripheral arteries and decreased vascular resistance in the lower extremities. Clinically, patients reported warmer lower limbs, less cold sensation, better walking tolerance and less numbness or soreness after treatment. Some older patients who had previously experienced nighttime coldness or discomfort in the legs also reported symptomatic relief.

### Enhancement of sleep quality

Treatment resulted in a significant improvement in sleep quality as measured by the Pittsburgh Sleep Quality Index (PSQI). Most of the patients had symptoms related to insomnia before therapy, including prolonged sleep latency, frequent night awakenings, shallow sleep, early morning awakening, fatigue in the daytime, irritability and decreased concentration during the daytime. The mean PSQI score decreased significantly after treatment from 14.25 ± 3.12 to 7.86 ± 2.45 (P < 0.01), indicating marked improvement in sleep quality. Improvement in sleep quality following treatment is illustrated in Figure 5.

**FIGURE 5:**
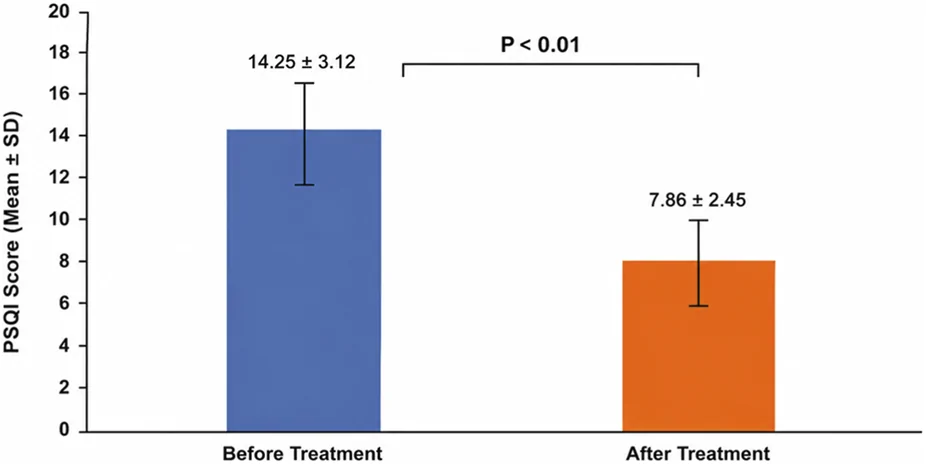
Enhancement of sleep quality following TCM foot bath preparation. Comparison of Pittsburgh Sleep Quality Index (PSQI) scores before and after treatment demonstrated a significant reduction in mean PSQI values following therapy, indicating substantial improvement in sleep quality and sleep-related symptoms among patients with lower limb oedema and sleep disorders. Values are expressed as mean ± standard deviation (SD) (P < 0.01).

After treatment, many patients reported easier sleep initiation, longer sleep duration, fewer awakenings at night, and better energy during the day. Some patients also reported reduced emotional irritability, increased mood stability and enhanced relaxation after the foot bath sessions.

### Comparison of outcome measures pre- and post-treatment

The statistical analysis showed a highly significant improvement in the ankle circumference and the PSQI scores after the treatment. The ABI values also showed significant improvement as compared to the baseline values.

The primary interpretation of treatment-associated changes was based on the three predefined quantitative outcomes: ankle circumference, ABI, and PSQI. The reduction in ankle circumference represented a measurable change in lower-limb swelling, the increase in ABI represented a measurable change in peripheral arterial perfusion, and the reduction in PSQI represented a structured improvement in sleep quality. The supportive clinical response categories and total effective rate were interpreted only as descriptive clinical information and were not used as independent validated efficacy endpoints. Because this was a retrospective single-arm study, these findings should be interpreted as associations observed after treatment rather than proof of causal efficacy.

### Safety assessment and adverse reactions

Safety outcomes were evaluated using the adverse-event information available in the clinical records. Mild local adverse events, including transient skin redness, mild dryness, and temporary warmth sensation, were documented in approximately 2% of patients. These events resolved spontaneously or improved following topical emollient use, adjustment of the water temperature, or brief treatment modification. Among the analyzed records, no treatment discontinuation was attributed to an adverse event and no thermal burns or severe cutaneous injuries were documented. However, because adverse-event information was collected retrospectively and the analysis included only treatment completers with complete records, adverse events and treatment discontinuations may have been incompletely ascertained.

## Discussion

The present retrospective study described pre-treatment and post-treatment clinical outcomes of a TCM foot bath preparation in 2000 patients with lower limb oedema accompanied by sleep disturbance. The results showed that the ankle circumference, the peripheral circulation (measured by ABI), and sleep quality (measured by PSQI scores) after treatment were significantly improved. These findings describe pre-treatment and post-treatment differences in the analyzed records; the uncontrolled retrospective design prevents conclusions regarding causality, efficacy, or effectiveness. The link between the traditional rationale and the clinical outcomes was established at the level of outcome selection and interpretation rather than direct mechanistic proof. In Traditional Chinese Medicine theory, lower-limb swelling, heaviness, coldness, and discomfort are commonly interpreted in relation to dampness retention, impaired movement of Qi and blood, and blood stasis. In the present study, these traditional symptom domains were mapped to measurable clinical variables: ankle circumference was used to quantify swelling, ABI was used to evaluate peripheral circulation, and PSQI was used to evaluate sleep quality. Therefore, the study did not rely solely on traditional symptom descriptions but assessed whether the intervention was associated with changes in predefined clinical measures. However, mechanistic explanations based on blood activation, dampness removal, thermal stimulation, or meridian effects remain theoretical and should be confirmed in future controlled studies using objective mechanistic assessments.

From a TCM perspective, the intervention was selected to activate blood circulation, resolve stasis, promote dampness elimination, and calm the Shen, which provides the traditional rationale for its use in oedema and sleep disturbance.

The botanical formulation for this study was comprised of the stir-fried peach kernel (Tao Ren), bush cherry seed (Yu Li Ren), Chinese angelica (Dang Gui) and safflower (Hong Hua). The prescription was made according to the classic TCM theories of activating blood and removing stasis, promoting urination, nourishing blood and calming the mind (Cheng et al., 2017). Because this retrospective study did not include mechanistic biomarkers, vascular imaging, inflammatory markers, autonomic function testing, pharmacokinetic assessment, actigraphy, or polysomnography, the proposed mechanisms should be interpreted as theoretical explanations based on TCM principles and previously published biomedical literature rather than mechanisms directly demonstrated by the present dataset.

In Traditional Chinese Medicine (TCM), peach kernel and safflower are considered classic blood-activating botanical drugs, often used for blood stasis and impaired circulation-related disorders (Su et al., 2021). Recent pharmacological studies indicated that extracts of peach kernel may possess the effects of inhibiting platelet aggregation, decreasing blood viscosity, improving hemorheological properties and improving microvascular perfusion. Safflower contains bioactive metabolites, such as safflower yellow pigments and flavonoids with vasodilatory, antioxidative, anti-inflammatory, and anticoagulant properties. The combination of peach kernel and safflower thus may improve peripheral blood circulation through a variety of mechanisms, including reduction of vascular resistance, enhancement of endothelial function, improvement of capillary perfusion and acceleration of venous return. This is because better circulation reduces local tissue congestion and capillary hydrostatic pressure, allowing for the reabsorption of interstitial fluid and reducing oedema.

Chinese angelica is traditionally used to nourish and invigorate blood, and its reported vascular and sedative-related properties may theoretically support circulation and sleep; however, these effects remain hypothesis-generating in the present study.

Bush cherry seed (Yu Li Ren) has traditionally been used to moisten the intestines and promote urination (Su et al., 2021). The classical TCM literature also reports on its efficacy in reducing oedema and facilitating the elimination of dampness accumulation. In the present study, bush cherry seed may participate in regulating fluid metabolism and reducing swelling in lower limbs. From a modern physiological point of view, the promotion of urination and improved fluid elimination may, at least in part, reduce the accumulation of extracellular fluid and tissue oedema. The botanical drug may also benefit lower limb comfort and circulation by aiding the removal of retained fluids and metabolic waste products. Apart from the pharmacological properties of the botanical formulation, the procedure of foot bath may also be of great importance for the therapeutic efficacy. According to TCM theory, the feet have many capillary networks, nerve endings and meridian channels which are related to many internal organs (Su et al., 2021).

The immersion in warm water at 40 °C–42 °C causes vasodilation and improves the peripheral blood flow in the lower extremities (Charkoudian, 2010). Heat exposure relaxes the vascular smooth muscle, decreases the peripheral vascular resistance and improves the capillary circulation. Increased venous return decreases venous congestion and capillary hydrostatic pressure, allowing reabsorption of interstitial fluid and resolution of oedema. The observed reduction in ankle circumference after treatment may be consistent with improved local fluid balance and reduced lower-limb swelling. However, this finding does not directly demonstrate the physiological mechanism of thermal therapy. Potential effects of warm-water immersion on endothelial function, microvascular responsiveness, and peripheral circulation should be considered hypothetical in the present study, because these mechanisms were not directly assessed. Endothelial dysfunction is increasingly recognised as a major contributor to chronic circulatory disorders and the development of oedema (Ja et al., 2017). Improved endothelial activity can improve vascular elasticity and efficiency of circulation, leading to long-term symptom improvement. Possible local exposure to botanical metabolites remains theoretical because transdermal absorption was not measured (Yang et al., 2022).

Stimulation of the foot region, including the SP6 area, may theoretically contribute to relaxation and sleep improvement, but autonomic or neurovascular mechanisms were not directly assessed. The improvement of PSQI scores can partly be due to the reduction of physical discomfort, improvement of lower limb circulation and psychological relaxation from the treatment procedure (Wen et al., 2020; Chang et al., 2025).

The present study showed that peripheral circulation and sleep quality improved at the same time, indicating that vascular function and sleep regulation are closely related. The simultaneous improvement in ABI and PSQI suggests a possible association between peripheral circulation and sleep quality, but this relationship remains observational and requires confirmation in controlled studies (Irwin, 2015; Mullington et al., 2009).

Previous clinical studies have reported the beneficial effects of TCM foot bath preparation for diabetic peripheral neuropathy, chronic venous insufficiency, insomnia, fatigue syndromes, and rehabilitation medicine (Song et al., 2023; Yang et al., 2010). However, many previous studies were limited by small sample sizes or mainly focused on single clinical outcomes. The present study provides a large descriptive dataset of observed pre-treatment and post-treatment differences in 2,000 patients over 5 years. Still, it does not provide controlled evidence of efficacy, effectiveness, or safety. Post-treatment ABI values were higher than baseline values; however, this difference cannot be attributed to the intervention because the study lacked a control group and adjustment for confounding (Abraham et al., 2012). Safety is a major consideration in the long-term management of chronic disorders of the circulatory system and insomnia. In the present study, mild local adverse reactions such as transient redness or dryness were seen in about 2% of the patients. Serious systemic adverse events or treatment-related complications were not observed. TCM foot bath preparation has several practical advantages over long-term pharmacological therapies, such as simplicity, low cost, minimal invasiveness and favourable tolerability (Tia et al., 2024). It is readily integrated into rehabilitation programs and chronic disease management strategies. However, caution is still needed in elderly, diabetic or sensory-impaired patients due to an increased risk of thermal injury (Kazamel et al., 2021). Appropriate temperature control and supervision are needed for safe treatment administration. In addition to temperature-related safety, potential botanical-drug-related risks should be considered. Persicae Semen and Carthami Flos are traditionally classified as blood-activating botanical drugs; therefore, patients with a bleeding tendency, pregnancy, severe vascular disease, or concomitant use of anticoagulant or antiplatelet medication require careful assessment. Although only mild local adverse reactions were recorded in this retrospective study, the absence of systematic drug-interaction assessment limits definitive safety conclusions.

This study has a number of limitations. First, the retrospective design and the single-center approach are uncontrolled and limit causal inference. Due to the lack of randomisation, blinding, a placebo group and a standard-care comparator, the improvements cannot be solely attributed to TCM foot bath preparation. The effects of natural fluctuations in symptom levels, placebo effects, expectation bias, regression to the mean, concurrent treatment, and lifestyle-related considerations may have affected the outcomes. Second, the diagnostic criteria were mainly derived from the routine clinical and TCM-related records and not fully internationalized diagnostic classifications. Furthermore, ankle circumference was chosen as a practical clinical measurement of lower limb swelling, and the total effective rate was only an optional descriptive variable, as it is not an internationally validated endpoint. The non-validated response classification, unblinded assessment, complete-case selection, uncontrolled co-interventions, and potential confounding introduce substantial risks of bias and may overestimate favorable outcomes. Hence, the primary interpretation should be based on quantitative results such as ankle circumference, ABI and PSQI.

Third, specific information about co-medications, compression therapy, rehab, physical activity, diet, disease severity, and other potential confounding variables was not always available. It could not be used to perform adjusted multivariable analysis reliably. Fourth, there was no long-term follow-up data available, and therefore, it is unclear whether there is long-term durability after treatment completion. Fifth, the study did not involve vascular imaging, endothelial or inflammatory biomarkers, autonomic function testing, pharmacokinetic assessment, actigraphy, or polysomnography, despite the fact that these may have provided additional information regarding possible mechanisms. Thus, mechanistic explanations should be viewed as theoretical and hypothesis-generating in nature and not directly proved by the present data set. Finally, adverse reactions were documented in regular clinical practice. Still, in future studies, it is necessary to use a unified adverse-event definition, adverse-event severity grading, causality assessment, and follow-up monitoring. In addition, although the botanical preparation was described using ConPhyMP-based reporting fields, complete batch-specific chemical fingerprinting and quantitative marker-metabolite assay data were not available for all historical preparations, which limits the reproducibility and pharmacological interpretation of the retrospective findings.

Future studies should be prospective, multicenter, randomized controlled trials with the use of adequate controls, blinded outcome evaluation if possible, uniform diagnostic criteria, extended follow-up, objective vascular and sleep measurements, systematic documentation of potential confounding variables, and thorough safety analysis. More diverse populations and healthcare settings, as well as patients with common co-morbidities under appropriate supervision, are also warranted studies. It is important to assess patient-reported experience, adherence and cost-effectiveness to elucidate the practicality of TCM foot bath preparation in rehabilitation and chronic disease management.

## Conclusion

This retrospective uncontrolled study identified post-treatment differences in ankle circumference, ABI, and PSQI among treatment completers. These observations do not establish efficacy, effectiveness, or definitive safety. Prospective controlled studies using validated outcomes and blinded assessment are required.

## Research integrity and transparency statement

The authors confirm that the study was conducted and reported in accordance with principles of research integrity, ethical transparency, accurate data reporting, and reproducibility. The retrospective design, absence of randomization and control group, incomplete availability of historical batch-specific quality-control information, and lack of long-term follow-up have been clearly acknowledged as study limitations.

## Data Availability

The original contributions presented in the study are included in the article/Supplementary Material further inquiries can be directed to the corresponding author.
