# Supplementary material for: Clinical outcomes following a traditional Chinese medicine foot bath preparation for lower-limb edema and sleep disturbance: a single-center retrospective study of 2,000 patients
Source: Front Pharmacol. 2026 Sep 22;17:1884794. doi: 10.3389/fphar.2026.1884794 (PMC13655585; doi:10.3389/fphar.2026.1884794)
Supplement: Supplementary file 1 [file Table1.docx]

**Supplementary Tables**

**Supplementary Table S1. Botanical preparation, extraction/process, pharmaceutical parameters, and quality-control documentation**

| **Reporting domain** | **Specific parameter** | **preparation** |
| --- | --- | --- |
| Preparation identity | Preparation type and route/use | Polybotanical aqueous decoction used externally as a Traditional Chinese Medicine foot bath for patients with lower-limb edema and sleep disturbance. It was not reported as an oral, injectable, or systemic preparation. |
| Composition | Botanical drug composition per treatment batch | Total crude botanical drug input: 45 g, consisting of Persicae Semen 10 g, Pruni Semen 15 g, Angelicae Sinensis Radix 10 g, and Carthami Flos 10 g. |
| Extraction medium | Solvent/extraction medium | Water. The botanical drugs were soaked in 2000 mL of water for 30 min before decoction. |
| Processing/extraction | Decoction procedure | The mixed botanical drugs were placed in a cloth bag, soaked in 2000 mL of water for 30 min, boiled on high heat, and then simmered for approximately 20 min until about 1500 mL of aqueous decoction remained. |
| Final use form | Final volume, dilution and temperature | Approximately 1500 mL decoction was transferred to a foot-bath basin and diluted/adjusted with warm water to maintain a therapeutic foot-bath temperature of 40–42°C during use. |
| Pharmaceutical parameter | Drug–extract ratio / crude-drug concentration | Approximate crude botanical drug input-to-decoction ratio before dilution: 45 g/1500 mL = 30 mg/mL crude-drug equivalent; equivalently, 1 g crude botanical drug per 33.3 mL decoction. This is a crude-drug-equivalent value, not a dry-extract yield. |
| Pharmaceutical parameter | Extraction yield / dry residue | Extraction yield was not determined because this was a retrospective clinical study, and the botanical preparation was freshly prepared for immediate external use rather than manufactured as a standardized extract. |
| Batch reproducibility | Batch preparation and reproducibility | One treatment batch should be prepared using the same four botanical drugs, the same quantities, the same soaking time, the same water volume, the same decoction time, and the same final volume/temperature. Batch-to-batch consistency should be supported by supplier certificates, pharmacopoeial identity tests, and chemical fingerprints from representative batches. |
| Storage/stability | Storage and stability conditions | The decoction should be described as freshly prepared for the day of treatment, used after cooling/dilution to 40–42°C, and not stored long term unless stability data are available. |
| Pharmacopoeial quality | Pharmacopoeial compliance | Each botanical drug material should comply with the relevant Chinese Pharmacopoeia monograph or equivalent institutional standard for identity, purity, foreign matter, moisture, ash/extractives, marker content, and absence of visible deterioration. Actual certificate-of-analysis values should be inserted from supplier/laboratory records. |
| Taxonomy | Taxonomic validation | Family names: Prunus persica (L.) Batsch / Prunus davidiana (Carrière) Franch. [Rosaceae]; Prunus humilis Bunge / Prunus japonica Thunb. / Prunus pedunculata (Pall.) Maxim. [Rosaceae]; Angelica sinensis (Oliv.) Diels [Apiaceae]; Carthamus tinctorius L. [Asteraceae]. |
| Authentication | Authentication procedure | Botanical drug materials were authenticated by qualified pharmacists in the Department of Pharmacy of Lanzhou Second People's Hospital according to the Chinese Pharmacopoeia (2020 Edition) based on macroscopic and pharmacopoeial identification. |
| Voucher records | Voucher specimens and depository | Voucher specimens were not retained because this was a retrospective clinical study using routinely supplied hospital botanical drug materials. |
| Chemical profile | Recommended orthogonal fingerprinting methods | Profile: (1) TLC/HPTLC fingerprinting for rapid identity comparison of botanical drugs; (2) HPLC-DAD or UPLC-DAD quantification/fingerprinting of polar and semi-polar marker metabolites; and (3) LC-MS/MS or UHPLC-QTOF-MS profiling for broader metabolite confirmation. GC-MS may be additionally used for volatile/phthalide-rich fractions, especially Angelicae Sinensis Radix, if available. |
| Marker/metabolite panel | Suggested marker/metabolite panel | Persicae Semen: amygdalin. Pruni Semen: amygdalin/prunasin. Angelicae Sinensis Radix: ferulic acid and Z-ligustilide. Carthami Flos: hydroxysafflor yellow A and safflower yellow pigments/carthamin-related markers. |
| Purity and contaminants | Contaminant/purity testing | Recommended tests include foreign matter, moisture/water content, total ash/acid-insoluble ash, extractives where applicable, heavy metals, pesticide residues, sulfur dioxide residues if relevant, aflatoxins/mycotoxins where required, and microbial limits. For an external foot bath, microbial quality and visible contamination should still be controlled to reduce skin-infection risk. |
| Safety monitoring | Safety monitoring during use | Skin was inspected before each session, and patients were observed for redness, burning, itching, dryness, dizziness, allergy, or thermal injury. Water temperature was maintained at 40–42°C, with extra caution for elderly, diabetic, and sensory-impaired patients. Mild transient local reactions were reported in approximately 2% of patients; no burns or severe cutaneous injury were reported. |
| Treatment regimen | Administration regimen | Once-daily external foot bath for 20–30 min/session. One course consisted of 10 daily sessions, and two consecutive courses were completed without a planned interval, giving 20 sessions over approximately 20 days. Feet were immersed up to the Sanyinjiao/SP6 level. |
